# When the Blue Marble Health concept challenges our current understanding of One Health

**DOI:** 10.1016/j.onehlt.2024.100935

**Published:** 2024-11-16

**Authors:** Marine Combe, Rodolphe Elie Gozlan

**Affiliations:** ISEM, Univ Montpellier, CNRS, IRD, Montpellier, France

**Keywords:** Global Health, Urban health, Neglected diseases, Dilution effect, Poverty

## Abstract

We address the issue of reconciling the hygienist and dilutionist (H&D) perspectives for a global understanding of health as envisioned in the One Health framework. Rich and poor countries share pockets of poverty on the outskirts of urban centres, known as ‘infectious bubbles’, which remain high-risk areas for disease emergence due to a common failure of both the H&D perspectives. People living in these IBs are exposed to infectious microbes on a daily basis due to inadequate hygiene infrastructure, while at the same time lacking a heathy nature to act as a buffer through a dilution effect. The Blue Marble Health approach shows that the burden of neglected diseases has also been neglected in rich countries. We argue for a single health framework that incorporates a mixed model of H&D views and addresses the issue of IB in the distribution and endemicity of emerging infectious diseases in large developed cities.

## 1 How to reconcile the hygienist and the One Health perspective?

**Fig. 1:**
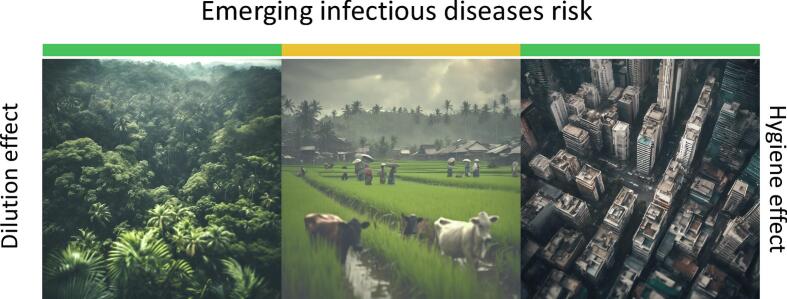
Current understanding of both the hygiene and dilution effect on the risk of emerging infectious diseases along an urban to pristine gradient, means that the dilution effect is the greatest in pristine natural ecosystem where biodiversity of competent and non-competent hosts is high while hygiene is the strongest in cities where people have access to clean water, clean food, an efficient cold chain, access to medicine and hospitals. The emerging infectious risk is thus relatively weak in pristine environments as there are few human and the biodiversity is high and in cities where the nature is mostly absent and people have access to high hygiene life style. Low emerging infectious disease risk is indicated in green; Mid emerging infectious disease risk is indicated in yellow. (For interpretation of the references to colour in this figure legend, the reader is referred to the web version of this article.)

The One Health approach, which integrates multi-sectorial experts and scientific disciplines, has become a popular framework for addressing health issues [1]. However, few have questioned the gaps emerging from this approach. Traditionally, health understanding was shaped by the Pasteurian model, leading to the “hygienist hypothesis,” which posited that reducing daily exposure to microbes would lead to health improvements. Despite advances in hygiene during the 20th century, the world has seen a sharp increase in emerging infectious diseases (EIDs) [2]. Many of these EIDs have been linked to environmental disturbances, such as climate change, deforestation, land use changes, and biodiversity loss [3]. The loss of biodiversity—caused by deforestation, urbanization, and agricultural intensification—has been suggested to increase EID risks in two ways: (i) by reducing non-competent hosts, which dilutes disease transmission, and (ii) by increasing interactions between competent hosts (disease carriers) and humans or domestic animals [[4], [5], [6]]. This theory, known as the “dilution effect,” implies that greater biodiversity can reduce infectious disease risk (Fig. 1) [7].

Since the 1980s, public health agencies have urged a broader view of human health that includes environmental and socio-cultural factors. In 2008, major organizations like the World Health Organization (WHO), the Food and Agriculture Organization (FAO), and the World Organization for Animal Health (OIE) officially embraced the One Health approach. The aim was to explore the relationships between the human, animal and environmental health sectors, especially concerning zoonotic diseases [3], and to foster global research initiatives and collaborations between the academic and private sectors as well as with societies. However, a central challenge for One Health is reconciling the hygienist hypothesis with the dilution effect. For instance, increasing urban biodiversity through reforestation or creating green spaces could boost human well-being but also raise short- and medium-term risks of “new” microbial infections. Humans might be exposed in these restored green spaces to microbes with which they co-evolved, potentially triggering regional epidemics or pandemics. Over the long term, however, this re-exposure could help populations regain immunity to these microbes [8]. Conversely, highly urbanized lifestyles, which limit exposure to environmental microbes, could weaken immune systems, leaving individuals more vulnerable when they do encounter these pathogens [9], such as through new urban nature pockets. Both scenarios—strict urbanization and increased urban biodiversity—could lead to imbalanced health outcomes. In urbanized settings, reduced exposure to diverse microbes may compromise immune systems, while the absence of biodiversity may foster environments where zoonotic pathogens may occur. For example, urbanization decreases the overall diversity of rodents but creates favorable conditions for species like *Rattus rattus* and *Suncus murinus*, which are highly competent carriers of zoonotic diseases, such as *Leptospirosis* and *Toxoplasmosis* [10]. The challenge for One Health is to strike a balance that maintains biodiversity while managing the risks of infectious diseases, especially in urban and developing environments.

## 2 The Blue Marble Health: a reality within the One Health perspective

The One Health approach, while aiming to address global health issues, often overlooks the complexity of health beyond the divide between developed (high-income) and less developed (middle- and low-income) countries. Health, according to WHO [11], is more than the absence of disease—it's a complete state of physical, mental, and social well-being. For example, intestinal parasitic diseases, which affect 1.4 billion people globally, are mostly documented in low-income countries [12]. However, this doesn't mean these diseases have disappeared from developed nations [13,14]. In the U.S., parasitic infections (Chagas disease, cysticercosis, toxocariasis, trichomoniasis) were historically common, but they are no longer seen as a threat for the Americans, even though they likely persist as is the case in Texas for instance [14]. Interestingly, the highest concentration of neglected diseases—often thought as tropical concerns—exists in the most developed countries, including the U.S., China, Japan, Germany and Brazil. For instance, schistosomiasis in Salvador in Brazil is an urban concern, and changes in parasite population is more likely due to improvements in living conditions (habitat, sanitation, etc.) rather than mass treatment with praziquantel [15]. Diseases like visceral leishmaniasis, schistosomiasis, hookworm, and Chagas disease remain highly prevalent in these G20 countries. In the U.S., at least 12 million people living in extreme poverty are believed to suffer from at least one of these neglected diseases [14]. Neglected diseases could also be linked to noncommunicable diseases, such as cancer, cardiovascular disease, and diabetes. For example, urogenital schistosomiasis is associated with an increased risk of HIV/AIDS [16], while hookworm infections can worsen malaria [17]. Chagas disease remains a major cause of cardiovascular problems, and liver flukes and schistosomiasis are linked to certain cancers [18] in wealthy countries [13,14]. These examples show that dividing health into “developed” vs. “developing” categories fails to reflect the interconnectedness of global health. EIDs are now shared across both developed and developing nations, further blurring this distinction [19]. In response to this changing landscape, the concept of “Blue Marble Health” (BMH) has emerged. BMH highlights that neglected diseases disproportionately affect the poorest communities, regardless of whether they live in wealthy or less developed nations [13,14]. While poverty is widespread in countries like Haiti, in wealthier countries like Brazil, Mexico and China, it is concentrated in “pockets of poverty.” This concept emphasizes that health disparities persist even within affluent nations.

**Fig. 2:**
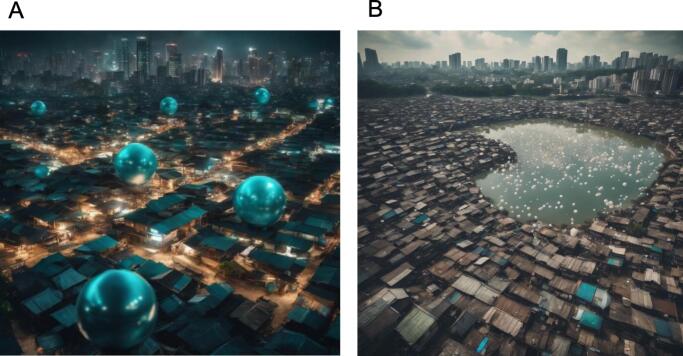
A: Blue Marble Health concept. The Blue Marble areas are areas of economic deprivation on the outskirts of towns and cities. Neglected diseases have become endemic in these « poverty pockets » [13,14] B: Infectious bubbles correspond to areas where the dilution effect is weak, because nature in these areas is highly degraded, although still present, and where poor populations with already degraded health have limited access to hygiene (clean water, cold food chain, sewage, etc.). (For interpretation of the references to colour in this figure legend, the reader is referred to the web version of this article.)

Urbanization and economic development tend to disrupt societies, creating gaps between the rich and poor. This divide, combined with the loss of biodiversity in increasingly sterile environments, weakens immune systems [20]. The poorest communities, living in “pockets of poverty” (Fig. 2A) [14], often find themselves at the intersection of urban centers (where hygiene dominates) and natural ecosystems (where the dilution effect might help). These areas, described as “infectious bubbles” (**IB**, Fig. 2B), are places where people are exposed to infectious microbes regularly. However, biodiversity in these areas is not sufficient to prevent disease transmission. The interface between these IBs and urban centers is crucial for understanding how diseases can spread. People living in these areas face increased risks of disease emergence, which could lead to regional epidemics or even pandemics. This highlights the need for a more integrated approach to health that recognizes the interconnectedness of human, animal, and environmental health across all economic divides.

## 3 How to integrate the BMH concept to our understanding of One Health?

**Fig. 3:**
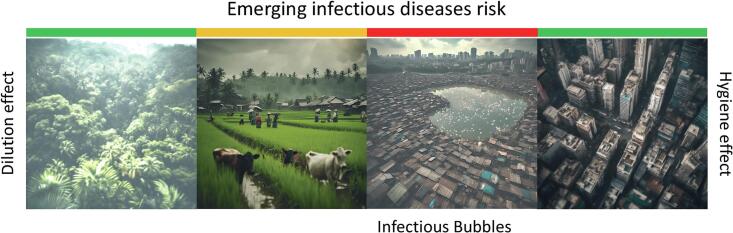
Impact of the hygiene and dilution effect on disease risk in different environments along a rural to urban gradient. The infectious bubbles are the areas where both the hygiene and dilution effects are weakest, resulting in a higher risk of emerging infectious disease. Green: low infectious risk; Yellow: mid infectious risk; red: high infectious risk. (For interpretation of the references to colour in this figure legend, the reader is referred to the web version of this article.)

The One Health approach integrates the environment, animal, and human health but overlooks the tension between the “hygienist” approach, focused on minimizing microbial contact, and the “dilutionist” approach, which values biodiversity's protective effects. This framework also fails to account for the spatial complexity of cities, where pockets of poverty persist alongside wealthy urban centers, creating areas with poor hygiene and insufficient biodiversity to trigger the dilution effect. These zones are particularly vulnerable to the spread of infectious diseases. Therefore, it is crucial to combine the concepts of hygiene and dilution to develop a comprehensive health strategy. By mapping where the gradients of hygiene and biodiversity intersect, we can identify high-risk health zones, which can be described as IBs. These areas, where low hygiene meets insufficient biodiversity, are small but significant breeding grounds for infectious diseases (Fig. 3). Integrating the BMH concept with One Health can help specifically target these IBs. Failing to incorporate BMH into One Health would ignore the neglected diseases still prevalent in wealthy nations and the role that poverty plays in the persistence of EIDs. Wealthy nations, especially G20 countries, must recognize their responsibility in addressing the diseases within their borders, particularly those exacerbated by economic inequality [21]. Economic development often creates these IBs, widening the gap between vulnerable and privileged populations and fostering conditions for disease emergence. The medical and scientific communities must recalibrate their understanding of One Health by incorporating BMH, acknowledging the delicate balance between poverty, IBs, and disease spread in urban centers. These IBs, and their critical role in disease emergence and transmission, should become a central focus of global health efforts within the One Health framework.

## 4 Perspectives

IBs are high-risk zones for disease emergence, making it crucial to reintroduce targeted biodiversity into cities. While global policies prioritize this, concerns remain about potential disease outbreaks. To address this, we propose merging the “hygienist” and “dilution” hypotheses into a combined model. Cities could integrate biodiversity zones that act as buffers, controlling disease by incorporating a specific balance of competent and non-competent hosts and vectors, rather than simply increasing overall biodiversity [22]. These zones would trap emerging pathogens and serve as sentinel areas for potential outbreaks. A meta-analysis showed that increasing non-competent hosts can reduce parasite infection rates [23]. For example, introducing the invasive non-competent snail *Lymnaea stagnalis* into ecosystems decreased trematode transmission stages (miracidia) in native competent snails [24]. Similarly, reductions were seen with nematode infections in native and introduced Hawaiian freshwater fishes [25]. This suggests that non-competent hosts might play a broader role in diluting various pathogens and their strains.

Governments, NGOs, and global health programs should recognize the infectious risks posed by IBs, where poverty is extreme, nature is degraded (low dilution effect), and hygiene is minimal (low hygiene effect). Developing strategies to create biodiversity zones that serve as dilution buffers should be a priority for urban and global health. These strategies should reduce disease burdens, create jobs, and benefit the global economy, ultimately contributing to poverty reduction. By integrating biodiversity zones into cities, we can reduce disease transmission while improving both public health and the environment, offering long-term economic and social benefits.

## Funding

This work was supported by the 10.13039/100010661H2020 Biodiversity Conservation to Mitigate the Risks of Emerging Infectious Diseases (BCOMING HORIZON-CL6–2021-BIODIV-01-11) and Preacts (PREZODE in action in the global South) programmes.

## CRediT authorship contribution statement

**Marine Combe:** Writing – original draft, Conceptualization. **Rodolphe Elie Gozlan:** Writing – review & editing, Writing – original draft, Conceptualization.

## Declaration of competing interest

The authors declare no conflict of interest. The manuscript has not been submitted or is not under review in another journal.

## Data Availability

No data was used for the research described in the article.
